# Examine the Moderating Role of Teacher’s Self-Efficacy in the Relationship Between the Job Satisfaction and Professional Learning Community in China

**DOI:** 10.3389/fpsyg.2022.841728

**Published:** 2022-10-03

**Authors:** Qiang-tian Li

**Affiliations:** School of Energy and Power, Jiangsu University of Science and Technology, Zhenjiang, China

**Keywords:** teachers’ self-efficacy, job satisfaction, COVID-19, professional learning community, China

## Abstract

**Abstract:**

**Purpose:**

The study aimed in examining the impact of the professional learning community, and teachers’ self-efficacy on the job satisfaction of teachers. Additionally, the study has also examined the moderating role of teachers’ self-efficacy in the relationship between the professional learning community (PLC) and job satisfaction.

**Method:**

The SEM-PLS is employed for the data analysis. The response rate of the study is 50%. The study is carried out on the primary teachers in China.

**Results:**

Three research questions were developed in the current study. The questions were related to the job satisfaction level of PE teachers, which was far beyond the level of satisfaction. As a result, teachers of PE classes had low performance. It was found by the study that professional learning community, job satisfaction of teachers, and self-efficacy are linked with each other. However, the results are inconclusive because of the limitation of the sample.

**Implications:**

The study has several implications among researchers, practitioners, and teachers.

**Significance:**

The study is among the few earlier studies on the issues related to Job Satisfaction and the professional learning community in China.

**Originality/Value:**

The study has highlighted an important issue related to the Job Satisfaction and professional learning community in China.

## Introduction

The spread of COVID-19 across the borders reflects that its impact cannot be stopped by anything. In order to prevent the spread of COVID-19, a policy of school closure was applied by Vietnam all across the country since February 4, 2020. A trilemma to ensure the continuation of learning progress, safety, and adequate standards of living for teachers was faced by the Vietnamese education system. Alternatively, there were limited threats of SAR-CoV-2 on teachers and students. Significant changes in the learning and teaching habits were observed because of the non-popularity of e-learning in the country (Tran et al., 2020; Sarfraz et al., 2021). Moreover, more than one million teachers in Vietnam improved their skills related to new technologies to deal with the current situation along with concerns for the future.

Some of the challenges and complexities have been highlighted because of changing learning process from physical to virtual space during COVID-19. The school system was not ready to adopt this way of learning and teaching. It was found by the students and teachers while studying and working from home that they are learning quickly about the use of digital tools (Dhawan et al., 2020; Palareti et al., 2020; Reimers et al., 2020). Lack of familiarity with these technologies resulted in several issues and difficulties in the required resources and ensuring access to education (European Commission (EC), 2020; Palareti et al., 2020).

It was also meant by this method that there may be a need for several devices and a suitable place at the same time to study or work (Cowden and Young, 2020; Filandri and Semi, 2020; Lucisano et al., 2020; Ranieri et al., 2020; Mohsin et al., 2021a; Naseem et al., 2021). Several teachers considered it as a source of stress to overcome these challenges in managing the distant learning process (Cowden and Young, 2020; Palareti et al., 2020; Ziebell et al., 2020).

It was argued by Mutsher (2018) that incentive is provided by professional learning community (PLC) for teachers to perform and become a motivation factor for students. Peers are allowed to share their feelings, belief, values, assessment, data analysis, discussion, and knowledge. Therefore, PLC should be built by teachers beyond their comfort zone and isolation (Hargreaves, 2019). For a new teacher to become professional, extra and longtime effort is required without active involvement in continuous professional development.

The adoption of PLC by a teacher in school makes him productive and active in sharing information, reading articles, delivering expertise to school, discussion of problems, visiting other schools, and making problem-solving strategies. Teachers have the advantage of becoming effective through this effort. Therefore, teachers become effective in their profession because of PLC. Active engagement was found among the PE teachers in professional learning community (PLC) with different teachers in the same schools and teachers from the same filed from other schools. In order to upgrade the skills of PE teachers, they were called to develop different forums for communication with colleagues within and out of the country for acquiring new knowledge (Mutsher, 2018).

It was found by the studies on productivity and job satisfaction that workers are productive when they are satisfied or happy (Mutsher, 2018; Lucas and LaRose, 2019; Gaturu and Njuguna, 2020). The job satisfaction level of PE teachers in China was really high (Lami, 2018). As a result, the teaching performance was low for PE classes. For this reason, most of the students did not attend PE classes because of a lack of interest and motivation (Trigueros et al., 2019; Seidu et al., 2020). There were many reasons for the low production level of PE teachers in terms of performance (Sinulingga and Simatupang, 2018). One of the key reasons behind the low performance was the incompetence of PE teachers in conducting classes of PE (Yilmaz et al., 2017). Teachers lack motivation for conducting classes. Moreover, they are unable to motivate students and attract them to performing their tasks (Abdullah et al., 2019). The incompetency of PE teachers is the main reason behind low performance in PE classes (Yilmaz et al., 2017). The accumulation of skills, knowledge and attitudes are the competencies. Self-efficacy is not possessed by PE teachers. The self-efficacy of teachers is regarded as to have confidence in their ability for the effective and successful conduct of teaching in any situation (Dolly et al., 2017). Teachers with high self-efficacy teach students by adopting new methods, strategies, and solutions. Students are inspired by self-efficient teachers, as they do not avoid complex situations. However, teachers with low self-efficacy tend to avoid complicated situations and blame others for their professional failure.

Different ways can be adopted for improving the self-efficacy of teachers. It has been suggested by theories that there is a need for providing competency courses to the teachers for increasing their level of self-efficacy. Teachers must be given feedback for their teaching by senior teachers or principals, which will assist them and help in sharing experience and knowledge (Dolly et al., 2017).

Professional learning community is one of the programs, which can help in improving the self-efficacy of teachers (Zonoubi et al., 2017; Suh and Dockery, 2018). Different actions are included in the PLC program, including knowledge diversity, teaching methods, skills, and professional adoption of new methods. The actions are carried out in a positive social environment with trust. Self-efficacy can be improved with the help of such an environment (Suh and Dockery, 2018). Schools are making efforts to offer learning programs for teachers in China. However, it has not been provided with reference to China that PLC can increase the self-efficacy of PE teachers, along with their level of job satisfaction. There is a lack of research on exploring the association between self-efficacy of PE teachers, job satisfaction, and PLC (Allami et al., 2017).

The previous research studies based on western economies suggest that the self-efficacy of teachers can be improved by PLC (Suh and Dockery, 2018). PLC influences the satisfaction level of teachers (Cervato and Kerton, 2017). However, there is a lack of research in this regard with reference to China. The theory of PLC was originated in the Western context. Thus, the issues linked with the relationship between the job satisfaction level of teachers, self-efficacy and PLC have not been conclusive. It was demonstrated by Hallinger et al. (2019) that theories must be tested by educational practitioners before applying them in any situation. With reference to developing economies, the issues linked with the association of self-efficacy of PE teachers, PLC and job satisfaction are unexplored and inconclusive. Therefore, there is a need for research to explore these issues.

## Hypothesis

The restrictions due to COVID-19 pandemic have imposed the integration of technology into teaching that requires teachers to adopt online teaching system. There is a difference of characteristics and qualities of learning and teaching experiences along with the context in the virtual classroom (Rice, 2006; Cho and Shen, 2013). Skills that are required by the teachers for teaching in the virtual environment are different from face-to-face interaction in a classroom. For instance, creation of a virtual environment, managing online classroom, motivation and encouraging students during online sessions, and maintaining the online environment and instructional design are some important skills (Kennedy and Archambault, 2012; Jackson and Jones, 2019). Further, there is a need for ensuring the presence of the learner in the online learning process (Garrison and Akyol, 2013; Anderson et al., 2017). The readiness of teachers in elementary and middle school was studied by Hung (2016) for teaching online. The researcher adopted the measure of readiness for online learning (TROLM).

Without the priorities of employees, working as an option from home was made mandatory due to COVID-19 crisis (Kniffin et al., 2021a,b). During the initial period of COVID-19, almost 80% of employees in half of the companies were working from home. This increased the trends of remote working (Gartner et al., 2020). Several employees were pressurized to work from home and challenges were faced by them because of basic issues. These issues included family size, living space, etc. (Kniffin et al., 2021a,b). Teachers were also among these employees who were forced to teach from home using online channels of communication. This was in line with the findings of Kniffin et al. (2021a,b). These measures and restrictions were made to prevent the outspread of the pandemic. It was documented by Kniffin et al. (2021b) that there is a difference in working from home before the pandemic and during the lockdown or quarantine. Before COVID-19, the preferences of employees and employers were involved in the decision of working from home. Further, it was based on the level of trust among the respective parties. However, the compulsion of working from home is different and unusual type of working from home.

The emergence of COVID-19 resulted in this new reality, which has influenced the job satisfaction of teachers. Teachers had to adopt alternative ways of working from 1 day to another during the pandemic. Teachers also have to work from home facing different challenges such as family, children, and household chores. However, it is not the same when a well-planned teleworking is done. The demands of a job are linked with unexpected telework that have resulted strains. All these factors have influenced the job satisfaction of teachers. However, the current research has been conducted based on the recent calls for research and the dedication of special issues related to COVID-19 in different journals.

A study was conducted by Zhang and Pang (2016) to compare the use of PLC between developing and developed countries. It was confirmed by the study that in developed countries, PLC is a compulsory element of the school system. Teachers come across several opportunities to acquire the latest knowledge and learn new strategies through effective communication with peer groups and teachers. Both learning and teaching are improved by PLC in developed countries. It was recommended by Thibaut et al. (2018) that teacher come across a broader vision regarding their methods, information careers by the use of PLC in schools. It creates a positive influence on organizational development.

The significance of using PLC in schools was examined by Zhang and Pang (2016) to explore its relationship with the satisfaction of teachers. The theory of Maslow was used by the researchers. It was found by Zhang and Pang (2016) that the use of PLC in schools is linked with the motivation level of teachers. It is explained by this study that teachers can improve their efficiency by reflecting their intrinsic and extrinsic needs. The influence of PLC on teachers’ feelings was discussed through job satisfaction. Several demands are encountered by teachers, including concerns related to the needs of students, fulfilling standards and execution of the educational strategies and plans.

Issues are faced by teachers, which cannot be controlled, such as lack of equipment, overcrowded classrooms, and lack of assistance from the staff and community. This results in the dissatisfaction of teachers. The teachers are offered opportunities by PLC to acquire new knowledge, adopt new strategies, assess students and learn class management techniques (Cansoy and Parlar, 2018). Teachers work in isolation in several schools, which makes them unaware of new information or knowledge. Moreover, they keep teachers under high pressure, which results in low satisfaction of job. It was revealed by Cansoy and Parlar (2018) that job dissatisfaction feeling is linked with isolation. Alternatively, job satisfaction is linked with the collaboration of teachers at the workplace.

The development of teachers is enhanced by satisfaction. The findings reveal that the use of PLC in schools creates an impact on teacher development. The implementation of PLC in schools is supported by this study for improving the knowledge and performance of teachers. Moreover, the dissatisfaction feeling is reduced. It was mentioned by Cervato and Kerton (2017) that businesses and industries use PLC to improve the skills and performance of their employees. The attention of policy makers in the field of education is aroused by the continuous improvement. It motivates them to adopt PLC in institutions. PLC has been used by this study as a systematic way for motivating teachers to work in a single group. Moreover, it helps in answering several questions, which are raised in periodic meetings by the members.

Professional learning community has been regarded as an endogenous variable for obtaining different results such as reduction of isolation, positive culture, developing and implementing new strategies of teaching. PLC enables teachers to increase their job satisfaction and retention of teachers. It was stated by Lami (2018) that a low level of satisfaction is possessed by teachers because of the lack of cooperation between teachers. It may be due to a lack of physical conditions, which support teachers in the process of teaching. The theory of Maslow and Harzzbarg mentioned that satisfaction could be improved by the provision of basic needs. Moreover, it helps in improving physical conditions and social relations as well. It was considered by the researchers that dissatisfaction arises when basic needs are not fulfilled. It was argued by Lavy and Ghanayim (2020) that the motivation level and self-esteem of teachers increases when they contribute to the process of decision making and performance appraisal. However, low self-efficacy and motivation are resulted by excluding a teacher from the decision making process. Thus, the job satisfaction level of teachers is reduced by these factors.

It was noticed by several researchers that teachers’ observation had given contradictory results. It was found that cultural emergence is not helpful in improving collaboration and job satisfaction decreases. It was argued by Lavy and Ghanayim (2020) that there are some intrinsic and extrinsic factors related to every job. The researcher claimed that there is a great influence of extrinsic factors (i.e., physical environment) on the level of teachers’ job satisfaction. This increased job satisfaction level results in an improved level of production and job satisfaction (intrinsic factors). A study was conducted by Mutsher (2018) in which job satisfaction affected the performance of teachers. Teachers were regarded as the backbone for educating future generations. The findings of the study revealed that the performance of teachers and job satisfaction is greatly influenced. It was found by the study that social support, physical environment, and salary of the teachers along with the social environment, are contributive factors for improved job satisfaction level. It was explained by Reeves et al. (2017) that the performance of teachers could be developed in social interactions and peer collaboration as it creates a great influence on the job satisfaction level.

A research was conducted by Mlyuka (2015) in which teachers were regarded as the backbone in the development of the world. Moreover, the researchers suggested that the performance of teachers is based on several factors, including job conditions, salary, number of students, attitude and personality toward work, etc. The job satisfaction variable plays a significant role in improving the performance of teachers. The sample of the study included 20 teachers from elementary school. The findings of the study showed that the social environment of the school has a significant effect in improving job satisfaction level and performance level of teachers.

Some survey studies were conducted by Gaturu and Njuguna (2020) in the sub-countries of Kenya. The sample was based on 290 teachers and 101 head teachers. The findings revealed using a two-factor theory, different factors affecting the level of job satisfaction of teachers were analyzed. The researchers found that poor working conditions, low incentives, lack of development opportunities, bad medical allowance, poor planning, an overload of work, lack of communication with peers are the factors, which negatively affect the satisfaction level of teachers. These factors must be reviewed for improving the performance and satisfaction of teachers in Kenya. In developed countries, schools focus on giving priority to school teachers. Teachers are the professionals who deliver their skills and knowledge to the upcoming generation. Moreover, the basic needs of the teachers must be fulfilled and prioritized by the authorities in educational planning for keeping them motivated (Nyagaya, 2015).

It was clarified by Mintrop and Ordenes (2017) that it is hazardous to overlook the feelings of teachers as their dissatisfaction is linked with a sharp reduction in performance. The researchers suggested that lack of commitment, isolation, absence, and alienation are linked with a feeling of dissatisfaction among teachers. However, the performance of teachers improves when they develop a sense of satisfaction.

*H1:* PLC has significant impact on the job satisfaction.

There are a lot of requirements and needs of teachers for consideration in daily routine while teaching students. Teachers become depressed with high work pressure and ambiguous goals, which results in low performance. The low performance results from a lack of guidance and support from their counterpart (Lucas and LaRose, 2019). It has been argued by researchers that there is a relationship between positive internal motivations, self-efficacy, and favorable performance with job satisfaction (Dugan et al., 2019). Moreover, these variables create an influence on a teacher’s willingness to work and his/her performance. A survey study was used to determine the influence of self-efficacy on the job satisfaction level of teachers (Kim and Seo, 2018; Zia-ur-Rehman et al., 2021). The results of the study revealed that there is a strong association between self-efficacy and job satisfaction. Thus, it was suggested that self-efficacy must be developed among teachers in order to improve their job conditions and motivation.

A study was conducted by Soto and Rojas (2019) based on a sample of 2000 teachers. It was found by the study that high self-efficacy level among teachers enables them to manage their classes effectively by adopting new teaching strategies, which influences their job satisfaction level. There is an influence of self-efficacy on the beliefs of individuals and the way in which priorities are arranged. This influences the behaviors of individuals as well (Dolly et al., 2017).

A key variable in creating the desired outcomes is positive self-efficacy. A positive self-efficacy has the power of catalyst for the teachers to deal with certain challenges. A teacher having positive self-efficacy is able to alter the ambient conditions to favorable ones. Therefore, job satisfaction is positively influenced by the self-efficacy of teachers.

Several studies reveal that the belief of teachers in self-efficacy has gained significant attention. The theory of Bandura originated this concept, which defines the feelings of a teacher in experiencing difficulties during the execution of his job. The definition of Bandura is related to a teacher’s performance that is able to achieve his goals by regulating his performance and behavior. A crucial role is played by self-efficacy in the professional life of a teacher (Kim et al., 2020). A high level of self-efficacy among teachers enables them to overcome challenges through good planning for achieving objectives successfully. Such teachers reflect an initiative spirit in their work. A crucial role is played by self-efficacy in the professional life of a teacher (Kim et al., 2020). A high level of self-efficacy in teachers enables them to deal with challenges through effective planning for achieving objectives successfully. Thus, when a teacher possesses a higher self-efficacy level, he is able to adopt good policies for dealing with difficulties. When a positive sense of self-efficacy is developed by a teacher, he becomes able to motivate his peers for improving various activities of the school.

It was claimed by Sahakyan et al. (2018) that there is a relationship between the self-efficacy of teachers and motivated expectations. The performance of teachers is improved through self-efficacy, and they become able to deal with challenges through the implementation of successful strategies and planning. The performance of students is overshadowed positively by this high self-efficacy feeling. It was noted by Soto and Rojas (2019) that teachers grow professionally by a high self-efficacy. There is a need for teachers to use different methods, strategies, materials in teaching students, which help students in achieving their educational obligations.

It was observed by Sahakyan et al. (2018) that a higher self-efficacy sense among teachers makes them open-minded. They are ready to learn modern strategies and ideas for growing professionally. Moreover, job satisfaction and self-satisfaction are developed by this attitude. It was stated that a high sense of self-efficacy is not influenced by the implemented and regulated courses of behavior. A teacher receives an intrinsic power to deal with several challenges for achieving his goals. It was expounded by.

Stephanou, Hallinger et al. (2019) that there is a significant influence of self-efficacy on the collective efficacy of a team because of cooperation between the members. In order to achieve educational obligations, a positive influence is created on job satisfaction by self-efficacy. Moreover, it was noted by Hallinger et al. (2019) that the collective efficacy of a team is shaped through self-efficacy. Moreover, it improves the job satisfaction level as well. The study has propped the following hypothesis:

*H2:* Teacher’s self-efficacy has significant impact on the job satisfaction.

*H3:* Teacher’s self-efficacy moderates the relationship between the PLC and job satisfaction.

## Methodology

To analyze the proposed hypotheses, we distributed 124 questionnaires for the purpose of data collection and testing of hypotheses. In present research, random sampling is chosen because it can resolve the issue of non-response rate and also minimizes errors (Hair et al., 2016; Richter et al., 2016; Li et al., 2021; Mohsin et al., 2021b). There is a significant relationship between the sample size and accuracy of the results, which means that accurate results are obtained when the sample size is large and errors can occur if small sample size is chosen for the study (Hair et al., 2017). Through random sampling the potential damages can be avoided (Ong and Puteh, 2017). Therefore, the main reason to choose oversampling is that it does not allow the non-response rate to affect the outcomes. However, in social science research survey the minimum response rate which is deemed acceptable is 50% (Hair et al., 2016). The items are taken from the prior studies.

The sample size of the current study is choose using the criteria laid down by Kyriazos (2018), according to which the sample size should be 300 as below that is roistered a weak sample. The study has chosen 620 as final sample size and out which the 319 questionnaire were usable. The response rate is turned out to be 51.40.

## Results

The study has used the SEM-PLS: A two-dimensional construct namely the measurement model, and structural model. The assessment of measurement model is a first step in SEM-PLS analysis.

**Figure 1:**
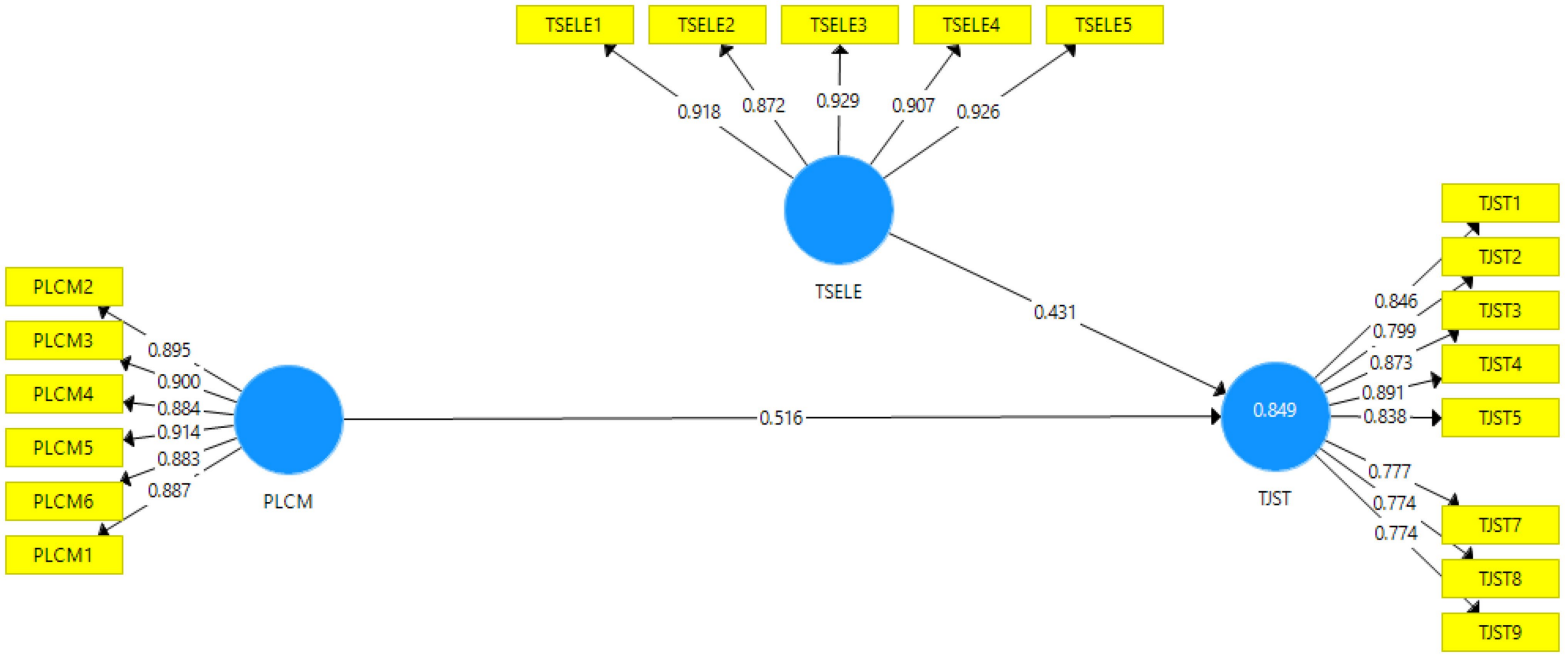
Measurement model.

To access the construct validity, discriminant validity, and convergent validity we have employed the measurement model. The measurement model is shown in the Figure 1.

The outer loading of the measurement model is shown in the Table 1. The item namely TJST6 with loading below 0.70 is deleted from the final analysis. Outer loading and cross loadings values are shown in the Table 1.

**Table 1:** Cross loadings.

	PLCM	TJST	TSELE
PLCM1	0.887	0.802	0.824
PLCM2	0.895	0.798	0.764
PLCM3	0.900	0.801	0.798
PLCM4	0.884	0.787	0.752
PLCM5	0.914	0.855	0.834
PLCM6	0.883	0.786	0.809
TJST1	0.777	0.846	0.796
TJST2	0.786	0.799	0.766
TJST3	0.808	0.873	0.797
TJST4	0.831	0.891	0.815
TJST5	0.800	0.838	0.852
TJST7	0.636	0.777	0.571
TJST8	0.624	0.774	0.598
TJST9	0.598	0.774	0.581
TSELE1	0.830	0.840	0.918
TSELE2	0.781	0.747	0.872
TSELE3	0.821	0.823	0.929
TSELE4	0.815	0.810	0.907
TSELE5	0.813	0.834	0.926

The reliability of measurement model is shown in the Table 2 below. The results confirm that there is no issue of reliability as all the values of each and every criteria are above the threshold values. The results of the reliability analysis are shown in the Table 2.

**Table 2:** Reliability.

	Cronbach’s alpha	rho_A	Composite reliability	Average variance extracted (AVE)
PLCM	**0.950**	**0.950**	**0.960**	**0.799**
TJST	**0.932**	**0.939**	**0.943**	**0.677**
TSELE	**0.948**	**0.950**	**0.960**	**0.829**

The meaning of the bold values is an “evaluation index” for different methods to compare the result.

The results of the validity matrix are shown in the Table 3. The results confirm that all the diagonal values are highest, thereby confirm the validity of matrix.

**Table 3:** Reliability analysis.

	PLCM	TJST	TSELE
PLCM	0.894		
TJST	0.891	0.823	
SELE	0.892	0.811	0.911

The *R*-square value is shown in the Table 4 below. The value is non-zero.

**Table 4:** *R*-square.

	*R* square
TJST	0.849

**Figure 2:**
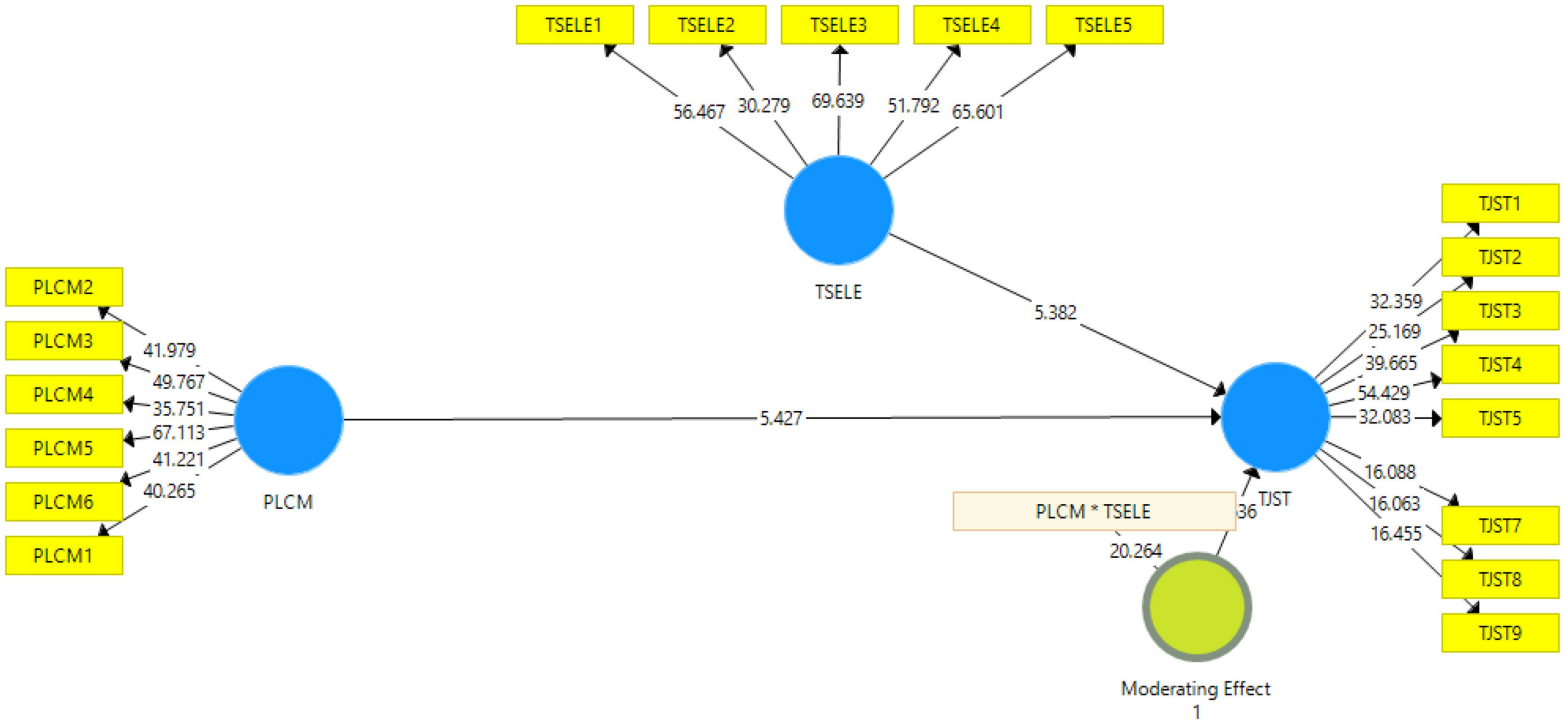
Structural model.

After the assessment of measurement model, the next step is the assessment of structural model. The structural model is shown in the Figure 2 and Table 5.

**Table 5:** Regression results.

	Original sample (O)	Sample mean (*M*)	Standard deviation (STDEV)	*T* statistics (|O/STDEV|)	*p* values
PLCM*TSELE → TJST	0.040	0.040	0.025	1.936	**0.000**
PLCM → TJST	0.468	0.463	0.086	5.427	**0.000**
TSELE → TJST	0.451	0.459	0.084	5.382	**0.000**

The meaning of the bold values is an “evaluation index” for different methods to compare the result.

The findings of the research reveal that the implementation of PLC, job satisfaction, and self-efficacy of teachers was low in the case of PE teachers of China during covid-19 The implementation of PLC was not properly done in the education system of China during covid-19, which made teachers feel dissatisfied and uncomfortable. There was a lack of planning and scheduling in the early days of covid-19 in China. Moreover, some teachers feel high pressure because of PLC implementation. Therefore, the job satisfaction and self-efficacy of teachers was influences passively by the implementation of PLC in China during covid-19. The acquisition of knowledge and gaining experiences was restricted. Most of the teachers had a passive level of self-efficacy, which resulted in the loss of planning and execution. The outcomes are the feeble achievements of students and the performance of teachers (Dolly et al., 2017; Naiwen et al., 2021). It was confirmed by Dolly et al. (2017) that individuals become unable to make decisions and share initiatives with others because of low self-efficacy level. When the self-efficacy level is low, there is high isolation, continuous failing, low job satisfaction, lack of sharing of PLC activities.

## Conclusion

Three research questions were developed in the current study. The questions were related to the job satisfaction level of PE teachers, which was far beyond the level of satisfaction (Lami, 2018). As a result, teachers of PE classes had low performance (Sinulingga and Simatupang, 2018). For this reason, most of the students lack interest in attending PE classes (Trigueros et al., 2019; Seidu et al., 2020). Self-efficacy was not possessed by the PE teachers. Professional learning community was one of the programs, which can be adopted for improving the self-efficacy of teachers (Zonoubi et al., 2017; Suh and Dockery, 2018). Attempts are made by schools to offer teacher learning programs in China. However, the implementation of PLC in China has not found to be improving the self-efficacy of PE teachers and job satisfaction level. There is a lack of research on exploring the relationship between job satisfaction of PE teachers and their self-efficacy of PE teachers in China (Allami et al., 2017).

### Recommendations for Future Studies

The aim of the study was to analyze the relationship between the self-efficacy of teachers, the professional learning community, and job satisfaction. Moreover, it was investigated whether the relationship between the job satisfaction of teachers and the professional learning community is mediated by self-efficacy or not. It was found by the study that professional learning community, job satisfaction of teachers, and self-efficacy are linked with each other. However, the results are inconclusive because of the limitation of the sample. The sample was based on PE teachers of four provinces, including (Allami et al., 2017). Therefore, it is recommended by the study that the current research should be replicated using different samples. A different questionnaire can be used in other replica studies for analyzing the relationship between job satisfaction, self-efficacy of teachers and the professional learning community.

## Data Availability Statement

The original contributions presented in the study are included in the article/supplementary material, further inquiries can be directed to the corresponding author.

## Ethics Statement

Ethical review and approval was not required for the study on human participants in accordance with the local legislation and institutional requirements. Written informed consent for participation was not required for this study in accordance with the national legislation and the institutional requirements.

## Author Contributions

The author confirms being the sole contributor of this work and has approved it for publication.

## Conflict of Interest

The author declares that the research was conducted in the absence of any commercial or financial relationships that could be construed as a potential conflict of interest.

## Publisher’s Note

All claims expressed in this article are solely those of the authors and do not necessarily represent those of their affiliated organizations, or those of the publisher, the editors and the reviewers. Any product that may be evaluated in this article, or claim that may be made by its manufacturer, is not guaranteed or endorsed by the publisher.
